# An aza-macrocycle containing maltolic side-arms (maltonis) as potential drug against human pediatric sarcomas

**DOI:** 10.1186/1471-2407-14-137

**Published:** 2014-02-27

**Authors:** Clara Guerzoni, Stefano Amatori, Luca Giorgi, Maria Cristina Manara, Lorena Landuzzi, Pier-Luigi Lollini, Aurora Tassoni, Mauro Balducci, Marco Manfrini, Loredana Pratelli, Massimo Serra, Piero Picci, Mauro Magnani, Vieri Fusi, Mirco Fanelli, Katia Scotlandi

**Affiliations:** 1 PROMETEO Laboratory, Section of Biomolecular Therapies, RIT Department, Istituto Ortopedico Rizzoli, Bologna 40136, Italy; 2 Experimental Oncology Laboratory, CRS Development of Biomolecular Therapies, IstitutoOrtopedico Rizzoli, Bologna 40136, Italy; 3 Molecular Pathology Lab. “PaoLa”, Department of Biomolecular Sciences, University of Urbino “Carlo Bo”, via Arco d’Augusto, 2, 61032 Fano (PU), Italy; 4 Department of Basic Sciences and Fundamentals, University of Urbino “Carlo Bo”, P.za Rinascimento, 6, 61029 Urbino (PU), Italy; 5 Department of Experimental, Diagnostic and Specialty Medicine, University of Bologna, Bologna, Italy; 6 Clinica Ortopedica e Traumatologica III, Istituto Ortopedico Rizzoli, Bologna 40136, Italy; 7 Clinical Pathology, Istituto Ortopedico Rizzoli, Bologna 40136, Italy; 8 Department of Biomolecular Sciences, University of Urbino “Carlo Bo”, via Saffi 2, 61029 Urbino (PU), Italy

**Keywords:** Sarcoma, Cancer therapy, DNA damage, Apoptosis, Macrocycles

## Abstract

**Background:**

Identification of new drugs against paediatric sarcomas represents an urgent clinical need that mainly relies on public investments due to the rarity of these diseases. In this paper we evaluated the *in vitro* and *in vivo* efficacy of a new maltol derived molecule (maltonis), belonging to the family of molecules named hydroxypyrones.

**Methods:**

Maltonis was screened for its ability to induce structural alteration of DNA molecules in comparison to another maltolic molecule (malten). *In vitro* antitumour efficacy was tested using a panel of sarcoma cell lines, representative of Ewing sarcoma, osteosarcoma and rhabdomyosarcoma, the three most common paediatric sarcomas, and in normal human mesenchymal primary cell cultures. *In vivo* efficacy was tested against TC-71 Ewing sarcoma xenografts.

**Results:**

Maltonis, a soluble maltol-derived synthetic molecule, was able to alter the DNA structure, inhibit proliferation and induce apoptotic cell death in paediatric sarcoma cells, either sensitive or resistant to some conventional chemotherapeutic drugs, such as doxorubicin and cisplatin. In addition, maltonis was able to induce: i) p21, p15 and Gadd45a mRNA upregulation; ii) Bcl-2, survivin, CDK6 and CDK8 down-regulation; iii) formation of γ-H2AX nuclear foci; iv) cleavage of PARP and Caspase 3. Two independent *in vivo* experiments demonstrated the tolerability and efficacy of maltonis in the inhibition of tumour growth. Finally maltonis was not extruded by ABCB1, one of the major determinants of chemotherapy failure, nor appeared to be a substrate of the glutathione-related detoxification system.

**Conclusions:**

Considering that treatment of poorly responsive patients still suffers for the paucity of agents able to revert chemoresistance, maltonis may be considered for the future development of new therapeutic approaches for refractory metastatic patients.

## Background

Sarcomas are uncommon and heterogeneous malignant tumours that arise from mesenchymal tissues, such as bone, cartilage or muscle. They account for around 1% of all human malignancies, thus being defined as rare diseases. Together with brain tumours, sarcomas are among the more frequent solid tumours in children and adolescents and have therefore high social impact [1]. Paediatric sarcomas include either tumours carrying fusion oncoproteins, generated by recurrent chromosomal translocations (i.e. Ewing sarcoma and alveolar rhabdomyosarcoma), or tumours lacking defined genetic alterations and characterized by complex karyotypes and genetic instability (i.e. osteosarcoma) [2]. Despite this genetic diversity, they share an aggressive natural history with rapid growth and marked tendency to form metastases. In the pre-chemotherapeutic era, when patients received surgery alone, survival rate was under 20% due to development of metastasis [3,4]. Since then, outcome for patients with rhabdomyosarcoma, osteosarcoma and Ewing sarcoma has improved dramatically thanks to intensification of treatment schedule, better supportive care and local therapy or use of risk stratification for rhabdomyosarcoma, [5-8]. However chemotherapy dose escalation implies higher rate of severe toxicity (infertility, cardiomiopathy) and increased risk of life-threatening late events, such as secondary malignancies [9,10]. With a larger population of long-survivors, scientific and patients’ associations are very sensitive to the need of therapy improvement in terms of reducing side effects and increasing quality of life, as well as potentiating efficacy of treatment in patients with metastatic disease. In this paper we explored the preclinical efficacy of two new-generation maltol-derived synthetic molecules named malten [N,N’-bis[(3-hydroxy-4-pyron-2-yl)methyl]-N, N’-dimethylethylendiamine] and maltonis [4(N),10 (N)–bis[(3-hydroxy-4-pyron-2-yl)methyl]-1, 7-dimethyl-1, 4, 7, 10 tetraazacyclododecane] [11,12]. Both chemical agents belong to the class of highly versatile molecules hydroxypyrones, that include compounds bearing anti-proliferative activities against a wide range of cancer cells, either alone or in combination with metals [13]. Particularly 3-hydroxy-2-methyl-4-pyrone (maltol) is a natural compound used in food, beverage, tobacco brewing and cosmetics for its flavour and antioxidant properties [14]. Maltol and its derivates were found to exhibit anti-neoplastic activity attributed to the formation of reactive oxygen species (ROS) as well as coordination properties towards metal ions [15-18]. For this reason, ligands containing maltol have been developed and exploited as new potential metal-based anti-tumor drugs [19]. Recently, the anti-cancer potential of malten, a molecule belonging to this class of poly-alkylamino-bis-maltolic compounds has been confirmed in different tumor histotypes [11,12]. Particularly the effect of malten was studied in eight different cellular neoplastic models derived from both hematopoietic and solid tumours such as cervix carcinoma, glioblastoma, pleural mesothelioma and alveolar rhabdomyosarcoma: the latter resulted to be the more sensitive histotype with an IC50 two or three folds lower than the other models [11]. Since few treatment options are amenable to sarcoma patients, we evaluated the efficacy of both malten and maltonis in a representative panel of patient-derived human rhabdomyosarcoma, osteosarcoma and Ewing sarcoma cell lines. *In vitro* and *in vivo* effects on tumor growth were examined.

## Methods

### Chemicals and synthesis of maltolic compounds

All chemicals and compounds were purchased from Sigma-Aldrich (St. Louis, MO, USA) at the highest quality commercially available. Malten and maltonis were synthesized as previously described [12]. The purity of maltonis, over 99%, was checked by elemental analysis, ^1^H and ^13^C NMR and mass spectra.

### DNA electrophoretic mobility assay and PCR inhibition assay

An amount of 500 ng of pLL3.7 plasmid DNA was incubated in 20 μl of 10 mM Tris-HCl (pH 7.4) in absence or presence of the reported concentrations of maltonis, malten and cisplatin (CDDP), for 2 hours at 37°C. After incubation, DNA was separated by 0.8% agarose gel electrophoresis (AGE) and then stained by ethidium bromide. An aliquot of the same mixture was diluted to 0.25 pg/μl and amplified (2 μl per assay) by real-time quantitative PCR (Q-PCR) as previously described [11] using the following sets of primers, designed with Primer Express software [20]:

pLLF1 (F1): 5′-AATACCGCGCCACATAGCAG–3′,

pLLF1a (F2): 5′-ATTCTGAGAATAGTGTATGCGGCG-3′,

pLLF1b (F3): 5′-CTCTTACTGTCATGCCATCCGTAAG-3′,

pLLF2 (F4): 5′-GTTGTCAGAAGTAAGTTGGCCGC–3′,

pLLF2a (F5): 5′-TTGCCGGGAAGCTAGAGTAAGTAG-3′,

pLLF3 (F6): 5′–GCTGCAATGATACCGCGAGAC–3′,

pLLR (R): 5′-GTGCACGAGTGGGTTACATCG–3′.

The reactions are characterized by a common reverse primer and are able to amplify the same plasmid region producing amplicons of different length (121, 179, 240, 301, 494 and 622 bp).

### Cell cultures and pharmacological treatment

A panel of cell lines representative of rhabdomyosarcoma, osteosarcoma and Ewing sarcoma were considered to evaluate malten and maltonis efficacy. Bone marrow or dental pulp derived normal human mesenchymal stem cells (h-MSC) were obtained from healthy donors or patients with benign bone lesions. After washings, cells were plated in α-MEM (Lonza, Verviers, Belgium), supplemented with 100 units/ml penicillin, 100 μg/ml streptomycin (Sigma-Aldrich, Saint Louis, MO) and 20% inactivated fetal bovine serum (FBS) (Lonza). Saos-2, U-2OS, SK-N-MC, and RD-ES were from the American Type Culture Collection, ATCC; the alveolar rhabdomyosarcoma cell lines SJ-RH30 and SJ-RH4 were provided by Dr. A. Rosolen (University of Padua, Padua, Italy) and Dr. D.N. Shapiro (St. Jude Children’s Hospital, Memphis, TN) [21]; Ewing sarcoma cell lines TC-71 and 6647 were kindly provided by T.J. Triche (Children’s Hospital, Los Angeles, CA); all other osteosarcoma (IOR/OS9 and IOR/OS10) and Ewing cell lines (LAP-35) were obtained from the Rizzoli laboratories and were previously described [22,23]. The RD/18 cell line is a clone of the commercially available human embryonal rhabdomyosarcoma cell line RD (Flow Laboratories), obtained at the Cancer Research Section, University of Bologna, Bologna, Italy [24]. Resistant variants of U-2OS and Saos-2 osteosarcoma cell lines were obtained by subsequent exposure to increasing concentrations of doxorubicin or cisplatin as previously described [25,26]. The most relevant mechanism of drug resistance in U-2OS/DX^580^ and Saos-2/DX^580^ is the increased DX efflux mediated by ABCB1 (MDR1) membrane transporter as consequence of both amplification and over-expression of MDR1 [25,27]. The major mechanism of CDDP resistance is the increase of both intracellular levels and enzymatic activity of glutathione-S-transferase P1-1 (GSTP1-1) and of, at a much lower extent, μ-class GST [26]. TC/DOXO8 was generated by transfection with an expression vector containing full-length MDR1 cDNA and selected in doxorubicin, thus achieving ABCB1-mediated increased DX efflux [28]. All cell lines have been tested for absence of mycoplasma contamination with MycoAlert (Lonza) -last control March 2013- and authenticated by STR analysis using genRESVR MPX-2 and genRESVR MPX-3 kits (serac, Bad Homburg, Germany). The following locus were verified: D16S539, D18S51, D19S433, D21S11, D2S1338, D3S1358, D5S818, D8S1179, FGA, SE33, TH01, TPOX VWA. Last control was performed in November 2012. Cells were cultured in a humidified atmosphere at 37°C in Iscove Modified Dulbecco’s medium, IMDM (Lonza) supplemented with 10% FBS, (Lonza) 1% penicillin-streptomycin. Malten and maltonis were dissolved in double-distilled water at the final concentration of 10 mM (stock solution), stored in aliquots at -80°C and diluted before use. Cells were exposed to malten or maltonis at the reported concentrations with subsequent administration every 24 hours. The GSTP1 inhibitor 6-(7-nitro-2,1,3-benzoxadiazol-4-ylthio)hexane (NBDHEX), kindly provided by Professor Anna Maria Caccuri, University of Rome “Tor Vergata”, Italy, was used in combination with maltonis (0.3-100 μM) or CDDP (1 μg-100 μg/ml) for 72 h at 0.3-0.75 μM [29].

### Cell growth inhibition, soft agar colony formation, apoptosis and cell cycle analysis

To assess cellular proliferation, MTT assay (Roche, Indianapolis, IN) was employed according to manufacturer’s instructions. Cells were plated into 96 well-plates (range 2,500-10,000 cells/well) in IMDM plus 10% FBS, or α-MEM plus 20% FBS. After 24 hours, various concentrations of malten and maltonis (0.3-30 μM), were added and cells were exposed up to 72 hours. Analysis of cell cycle was performed after 48 hours of treatment (FACSCalibur, Becton Dickinson, Italy) whereas apoptosis was assayed after 72 h with Mebcyto Apoptosis Kit (MBL International, Woburn, MA) according to the manufacturer instructions. Anchorage-independent growth was evaluated after seeding of 3,300-75,000 cells/dish in IMDM-10% FBS plus 0.33% agarose (SeaPlaque, FMC BioProducts, Rockland, ME) with a 0.5% agarose/IMDM-10%FBS underlay [30].

### RNA extraction and real time RT-PCR

Total RNA was extracted from TC-71 cells control and treated with maltonis (1-3 μM) after 72 hours of incubation using RNeasy Mini Kit (Qiagen) or TRIzol (Invitrogen). RNA was employed for Q-PCR evaluation assay (37 different genes simultaneously amplified in 100-well discs, Qiagen, Hilden, Germany) and analysis of data were performed as previously described, refer to Additional file 1 for the complete primers sets sequences [11]. Q-PCR of Gadd45α was performed with the following primers: Fw-Gadd45α: 5′-GACCCCGATAACGTGGTGTT-3′, Rv-Gadd45α: 5′-CCTGGATCAGGGTGAAGT -3′. GAPDH endogenous control was performed with Taqman assay # Hs99999905_m1 (Applied Biosystems, Foster City, CA). Samples were analyzed using an ABI Prism 7900 Detection System (Applied Biosystems), according to manufacturer’s instructions. Expression levels of target genes were normalized to that of glyceraldehyde 3-phosphate dehydrogenase (GAPDH), and the relative quantification analysis was performed on the basis of either 2-ΔΔCT and comparative quantitation methods.

### DNA laddering evaluation

The DNA laddering assay was performed as previously reported [31].

### Immunofluorescence and evaluation of nuclear fragmentation

After 24 hours of treatment with the reported concentrations of maltonis, cells were stained with anti-phospho-H2AX antibody (Cell Signaling, Danvers, MA,) and counterstained with DAPI as previously described [32]. Confocal images were acquired with Leica TCS SP2, magnification 63X (Wetzlar, Germany). For evaluation of nuclear fragmentation, cells were seeded in 60-mm petri dishes and 24 hours later treated with 1-3 μM of maltonis. 72 h after treatment, cells were fixed in methanol/acetic acid (3:1) for 15 min and stained with 50 ng/ml Hoechst 33258 (Sigma). Cells with three or more chromatin fragments were considered apoptotic. The percentage of nuclei showing fragments was calculated considering 1,000 nuclei.

### Immunohistochemistry

Sections (5 μm) from formalin-fixed, paraffin-embedded tumours xenografts were placed on poly-l-lysine–coated slides (Sigma). Avidin-biotin-peroxidase procedure was used for immunostaining, as previously described [33]. For morphological evaluation of nuclear alterations, samples were counterstained with Mayer’s haematoxylin and eosin (Sigma). Detection of Ki-67 was performed on sections pre-treated with a citrate buffer solution [0.01 mol/L citric acid and 0.01 mol/L sodium citrate (pH 6.0)] in a microwave oven at 750 W and stained with the MIB-1 primary antibody (1:100 dilution; Calbiochem-Novabiochem, San Diego, CA). TUNEL assay was performed with ApopTag® Plus Peroxidase in situ apoptosis kit (Merck Millipore, Billerica, MA) according to manufacturer’s instructions.

### Western blotting

Cells were lysed with phospho-protein extraction buffer (Merck Millipore) supplemented with protease-phosphatase cocktail inhibitor (Sigma). 40 μg total lysates were then resolved on a 10% or 15% Tris-HCl gel and immunoblotted with the following specific antibodies: anti-BAX (6A7) monoclonal antibody (Santa Cruz Biotechnology, Dallas TX), anti-p21 (H-164) polyclonal antibody (Santa Cruz Biotechnology), anti-PARP polyclonal antibody (Cell Signaling Technologies), anti-BCL2 (clone100) monoclonal antibody (Merck Millipore), anti-caspase 3 (8G10) monoclonal antibody (Cell Signaling Technologies), anti-GAPDH polyclonal antibody (Santa Cruz Biotechnology).

### In vivo evaluation of maltonis efficacy

To evaluate anti-tumour efficacy, athymic Crl:CD1-Foxn1 nu (referred to as nude) mice were purchased from Charles River, Italy. Five weeks old mice were injected subcutaneously (s.c.) with 7.5 × 10^6^ TC-71 cells/mouse to obtain tumours xenografts. When tumours started to be measurable (approximately 7 days after cell inoculation) mice were randomized in two groups: i) control (n = 9) and treated (n = 5) ii) control (n = 5) and treated (n = 4). Control group was treated with vehicle alone (PBS), treated group received maltonis daily intra-tumour for two subsequent cycles of 5 days. Treated mice were injected with: i) 20 mg/Kg maltonis in the first cycle and 40 mg/kg in the second one or ii) 40 mg/kg for both cycles. Tumour dimensions were measured twice weekly and tumour volume was calculated as π[√(a · b)]^3^/6 where “a” is the maximal tumour diameter and “b” is the tumour diameter perpendicular to “a”. Four days after the end of treatment mice were sacrificed and tumour samples were collected.

All animal experiments were performed according to Italian law 116/92 and European directive 2010/63/UE. Experimental protocols were reviewed and approved by the Institutional Animal Care and Use Committee (“Comitato Etico Scientifico per la Sperimentazione Animale”) of the University of Bologna, and forwarded to the Italian Ministry of Health.

### Serum glucose, urea, and transaminase levels

Control or maltonis-treated mice were analyzed to verify whether the maltol derived compound modulated serum levels of glucose or induced suffering at hepatic or systemic level. Before the sacrifice, mice were sampled for blood. Determination of serum concentration of glucose and other enzymes was done.

### Statistical analysis

Differences among means were analyzed using a two-sided Student’s T test. When data were not normally distributed, the nonparametric Mann-Whitney rank-sum test was used. Correlations between two variables were obtained by Pearson test; differences among percentages with Chi Square test. IC_50_ values were calculated with CalcuSyn software (Biosoft, Ferguson, MO).

## Results

### Maltonis synthesis, screening and biological effect on tumour cell lines and normal human mesenchymal stem cells

**Figure 1:**
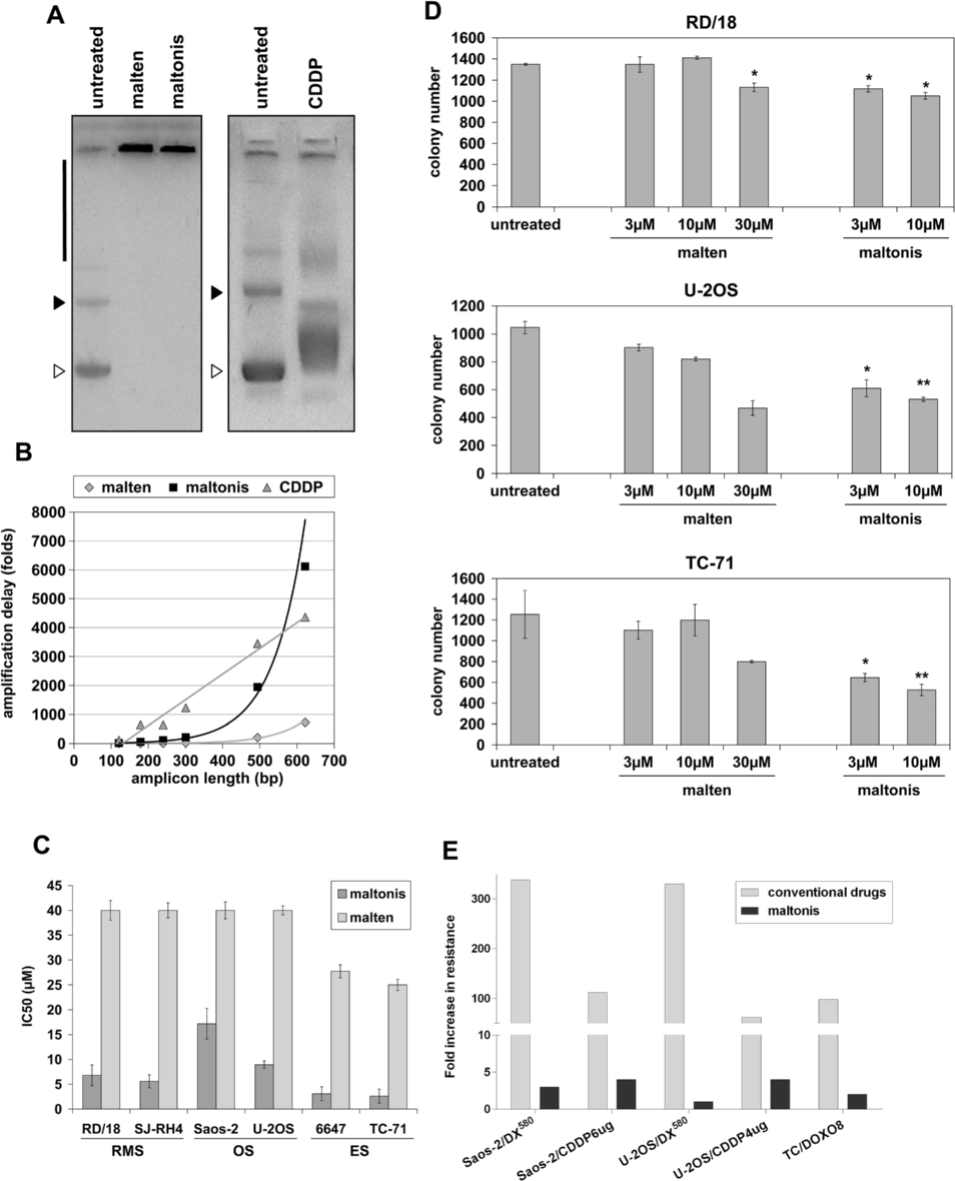
**Malten and maltonis effect on DNA structure and sarcoma cell growth. (A)** Effects of the two compounds on electrophoretic migration of plasmid DNA. After 2 hours incubation in presence of 4 mM malten, 4 mM maltonis or 25 μM CDDP, circular plasmid DNA (pLL3.7) was separated by agarose gel electrophoresis. Supercoiled (white arrow), open circular (black arrow) plasmid form and high molecular weight DNA complexes formation (black bar) are indicated. **(B)** PCR inhibition assay. Amplification delay (folds) was calculated for each set of primers (for details see Additional file 3) as the difference between the Ct values of treated and untreated samples. Graphical representation of the exponential decrease of the number of amplifiable DNA sequences after incubation with malten, maltonis or CDDP. **(C)** Histogram showing IC50 values for malten (light bars) and maltonis (dark bars) in a panel of sarcoma cell lines. RMS, rhabdomyosarcoma; OS, osteosarcoma; ES, Ewing sarcoma. Values are expressed as mean of three independent experiments ± SE. **(D)** Maltol derived compounds inhibit tumour growth in anchorage-independent conditions: columns are the mean of three independent experiments ± SE performed on RD/18 (seeded cells: 10,000/dish), U-2OS(seeded cells: 10,000/dish) and TC-71 (seeded cells: 3,300/dish) cells. Statistical analysis was performed by Student’s t test: *P < 0.05; **P < 0.01. **(E)** Fold increase in drug resistance to conventional drugs (light grey) or to maltonis (black): fold resistance is calculated on the values of IC50 of each resistant cell line against its respective sensitive parental one. Values are representative of three independent experiments.

Maltonis, a derivative of malten, (poly-alkylamino-bis-maltolic molecule) was obtained as a white solid perchlorate salt (L^.^3HClO_4_^.^H_2_O), stable at light, air and room temperature, and soluble in water (Additional file 2-see Materials and Methods) [11,12]. To evaluate its ability to interfere with the structure of nucleic acids, plasmid DNA was exposed to malten and maltonis and separated by agarose gel-electrophoresis: DNA treated with both compounds failed to migrate in agarose gel (Figure 1A, left panel). Moreover, we applied the recently developed PCR inhibition assay that detects covalent DNA modifications (Figure 1B) [11]. Cisplatin was used as control of known therapeutic molecule able to induce both single and double strand crosslinking [11]. Doubling the amplicon length, an exponential loss of PCR efficiency was observed in maltonis and malten treated DNA, while cisplatin (CDDP) treatment induced, as expected, a linear decrease of DNA amplificability (Figure 1B, Additional file 3) as consequence of the induced DNA modification (Figure 1A). The efficiency of delay appeared to be higher in maltonis- than in malten-treated samples. We next evaluated the efficacy of the two compounds against sarcoma cell growth. Two rhabdomyosarcoma, two osteosarcoma and two Ewing sarcoma cell lines displayed marked reduction of cell growth after 72 hours of treatment with maltonis (IC50 values ranging from approximately 3 μM to 18 μM) (Figure 1C); conversely malten showed modest efficacy only in the two Ewing cell lines (IC50 values between 25 μM and 40 μM). Similar results were obtained in anchorage independent conditions. Particularly after treatment with maltonis we observed a reduction of both the number and the size of colonies (Figure 1D, Additional file 4); whereas malten induced no significant impairment of growth. These data confirmed the therapeutic potential of maltonis but not malten in the management of sarcomas. The analysis of maltonis efficacy was extended to a larger panel of cell lines and to three human normal mesenchymal stem cells, considered the cell of origin of sarcomas (Table 1) [34-36]. Sensitivity varied in a range from 2.6 to 12.5 μM in patient-derived cell lines, without any remarkable difference either among the tumour histotypes nor in sarcomas carrying specific translocations or displaying complex genetic aberrations. Interestingly, IC50 values of maltonis in the different cell lines correlated to cell doubling times (r = 0.59, p = 0.01, Pearson correlation test), whereas human normal mesenchymal stem cells appeared to be unaffected by the compound. Moreover, maltonis was dramatically active either in cells resistant to multiple drugs (e.g. doxorubicin, epirubicin, vincristine) or resistant to cisplatin [25,26,28] (Figure 1E, Table 1). In particular, cells transfected with (TC/DOXO8) or overexpressing ABCB1/MDR1 gene (U-2OS/DX^580^, Saos-2/DX^580^) were sensitive to maltonis, indicating that the drug was not extruded by ABCB1 transporter. Cells resistant to cisplatin maintained very low levels of resistance to maltonis (around 4-fold) compared to parental cell lines, indicating that a partial cross-talk in the mechanisms of action between the two drugs may exist (Figure 1E). Since activity of glutathione S-tranferase P1 (GSTP1) was demonstrated to be relevant for cisplatin resistance of U-2OS and Saos-2 osteosarcoma cells, we tested the efficacy of maltonis in presence of the GSTP1 inhibitor 6-(7-nitro-2,1,3-benzoxadiazol-4-ylthio)hexane (NBDHEX) [26,29]. NBDHEX did not modulate the efficacy of maltonis but reduced the IC50 values of cisplatin as expected (Additional file 5), thus indicating that the glutathione-related detoxification system did not limit maltonis cytotoxic effect.

**Table 1:** Maltonis mean IC50 values in a panel of human derived sarcoma and human normal mesenchymal cells

**Histotype**^**a**^	**Cell line**^**b**^	**IC50 μM (± SE)**^**c,f**^	**Genetic alteration**^**d**^	**Doubling time (hours)**^**e,f**^
RHABDOMYOSARCOMA	RD/18	6.8 (± 2.1)	Altered 11p15.5 chromosome	22 ± 0.1
	SJ-RH4	5.6 (± 1.3)	(2;13)(q35;q14) PAX3 FKHR	25 ± 1.2
	SJ-RH30	9.9 (± 2.7)	(2;13)(q35;q14) PAX3 FKHR	26 ± 1.0
OSTEOSARCOMA	IOR/OS10	5.5 (± 1.3)	Complex Karyotype	30.4 ± 2.6
	IOR/OS9	8.6 (± 1.8)	Complex Karyotype	44.5 ± 2.4
	Saos-2	11.9 (± 3.1)	Complex Karyotype	36.1 ± 4.4
	Saos-2/DX^580^	39.3 (± 2.6)	Complex Karyotype	75.6 ± 5.7
	Saos-2/CDDP6ug	43.3 (± 6.8)	Complex Karyotype	44.3 ± 4.1
	U-2OS	12.5 (± 0.7)	Complex Karyotype	18.3 ± 0.2
	U-2OS/DX^580^	16.5 (± 0.4)	Complex Karyotype	45.3 ± 4.7
	U-2OS/CDDP4ug	56.1 (± 1.4)	Complex Karyotype	38.6 ± 4.1
EWING SARCOMA	6647	3.1 (± 1.4)	t(11;22)(q24;q12) EWSR1-FLI1 TYPE II	29 ± 2.3
	LAP-35	11.6 (± 0.7)	t(11;22)(q24;q12) EWSR1-FLI1 TYPE II	48.6 ± 4.2
	SK-N-MC	3.9 (± 0.5)	t(11;22)(q24;q12) EWSR1-FLI1 TYPE I	26.9 ± 0.5
	TC-71	2.6 (± 1.4)	t(11;22)(q24;q12) EWSR1-FLI1 TYPE I	14.9 ± 1.4
	TC/DOXO8	5.0 (± 0.1)	t(11;22)(q24;q12) EWSR1-FLI1 TYPE I	22.6 ± 2.8
h-MSC	MES1	>50	*n.d.*	*n.d.*
	MES2	>50	*n.d.*	*n.d.*
	DP-15	>50	*n.d.*	*n.d.*

^*a*^Histotype: origin of the patient-derived cell line.

^*b*^cell line name.

^*c*^mean IC50 calculated over three idependent experiments ± SE.

^*d*^genetic alterations associated to the cell line.

^*e*^doubling time of the indicated cell line.

^*f*^IC50 values statistically correlate with cell doubling times (r = 0.59, p = 0.01, Pearson correlation test).

*n.d.* not determined.

### Maltonis induces modulation of the gene expression profile

**Figure 2:**
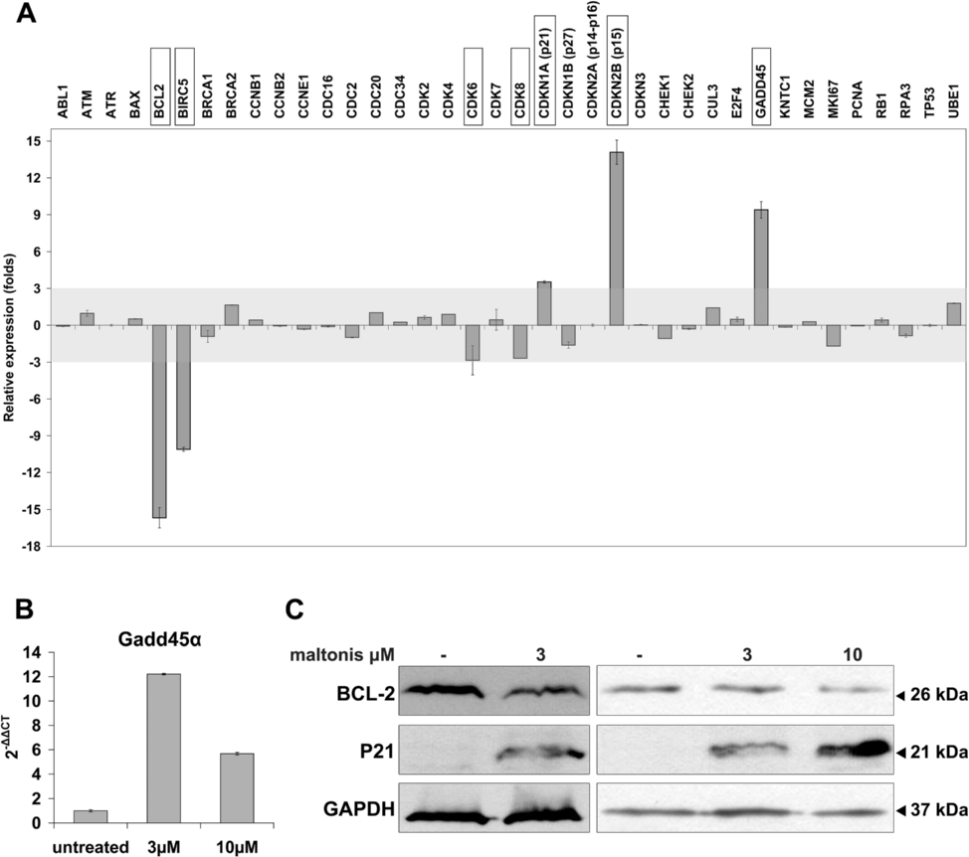
**Molecular response of TC-71 cells to maltonis exposure. (A)** Gene expression modifications induced by maltonis. Cells were exposed to maltonis for 48 hours at the concentration of 3 μM. Gene transcripts abundance was assessed by Q-PCR, normalized with GAPDH expression and evaluated as fold induction compared to untreated cells. Relative expression (folds) is reported as mean (± SD) resulting from three independent experiments. **(B)** Histogram shows relative PCR quantification of Gadd45-α mRNA (± SD) after 3 or 10 μM maltonis treatment. GAPDH expression was used as internal control; TC-71 untreated sample was used as calibrator. **(C)** Western blot evaluation of BCL-2, and p21 on total cell lysates from control or maltonis treated TC-71 cells. Equal loading was monitored with anti-GAPDH blotting (lower line).

To better define the molecular response triggered by maltonis, we evaluated the expression of genes known to regulate cell cycle progression, proliferation and apoptotic response by Q-PCR [11]. Exposure of TC-71 Ewing sarcoma cells to maltonis, at the dose of 3 μM for 48 hours, modified the transcript levels of some genes involved in the control of cell cycle progression: we monitored up-regulation of the cyclin-dependent kinase inhibitors (CDKI) CDKN2B (p15) and CDKN1A (p21), and down-regulation of cyclin-dependent kinase 6 (CDK6) and cyclin-dependent kinase 8 (CDK8) mRNA levels (Figure 2A). In addition, maltonis treated cells were characterized by a marked increase of Gadd45a mRNA levels and a strong down-regulation of survivin (BIRC5) and BCL-2 transcripts (Figure 2A). These genes were not regulated after treatment with malten [11]. The induction of Gadd45a mRNA was also confirmed by Q-PCR (Figure 2B). Two independent western blotting analysis confirmed that levels of protein expression were modulated consistently with gene profiling results: p21 were increased in TC-71 treated with maltonis, whereas the anti-apoptotic factor BCL-2 was diminished (Figure 2C).

### Maltonis induces perturbation of cell cycle progression in sarcoma cells

**Figure 3:**
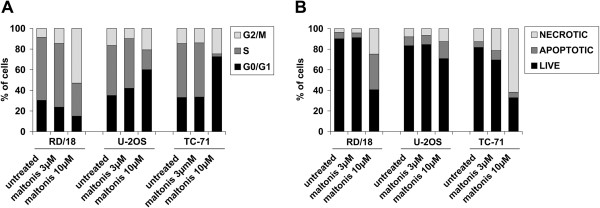
**Maltonis effect on cell cycle and apoptosis. (A)** Cell cycle evaluation of RD/18, U-2OS and TC-71 cells after 48 hours of treatment with maltonis 3 or 10 μM. Data are expressed as mean percentages of two independent experiments. Percentage of cells in the G1 or G2/M phase are significantly different after treatment compared to the respective controls (P < 0.01E^-16^ by Chi Square test for 10 μM). **(B)** Annexin V-PI analysis, expressed as percentage of live (black), apoptotic (dark grey) or necrotic (light grey) cells, in a panel of sarcoma after 72 hours treatment with 3 or 10 μM maltonis. Values are representative of 3 independent experiments. Percentage of apoptotic or necrotic cells are significantly different after treatment compared to the respective controls (P ≤ 0.02 by Chi Square).

The analysis of possible effects on cell cycle progression revealed the ability of maltonis to alter the cellular distribution in all the considered cell lines. Particularly we observed an accumulation of the rhabdomyosarcoma RD/18 cells in G2-M (Figure 3A) and of the osteosarcoma and Ewing sarcoma cells in G1 phase (Figure 3A); thus pointing out to the ability of this molecule to interfere with cell cycle progression as also confirmed by the modulation of CDKIs and CDKs (Figure 2A-C).

### Maltonis induces DNA damage and promotes apoptosis of sarcoma cells

Since the gene expression profiling and western blotting validations (Figure 2) suggested that an impairment of the pro/anti-apoptotic balance might be triggered by maltonis, we investigated RD/18, U-2OS and TC-71 cell death after exposure to the maltol derived compound. Flow cytometry analysis of AnnV-PI stained control and treated cells at 72 hours revealed that death occurred in all the three histotypes with different degrees (Figure 3B): TC-71 and RD/18 appeared to be the more sensitive to maltonis induced cell death, whereas U2-OS were only partly affected.

**Figure 4:**
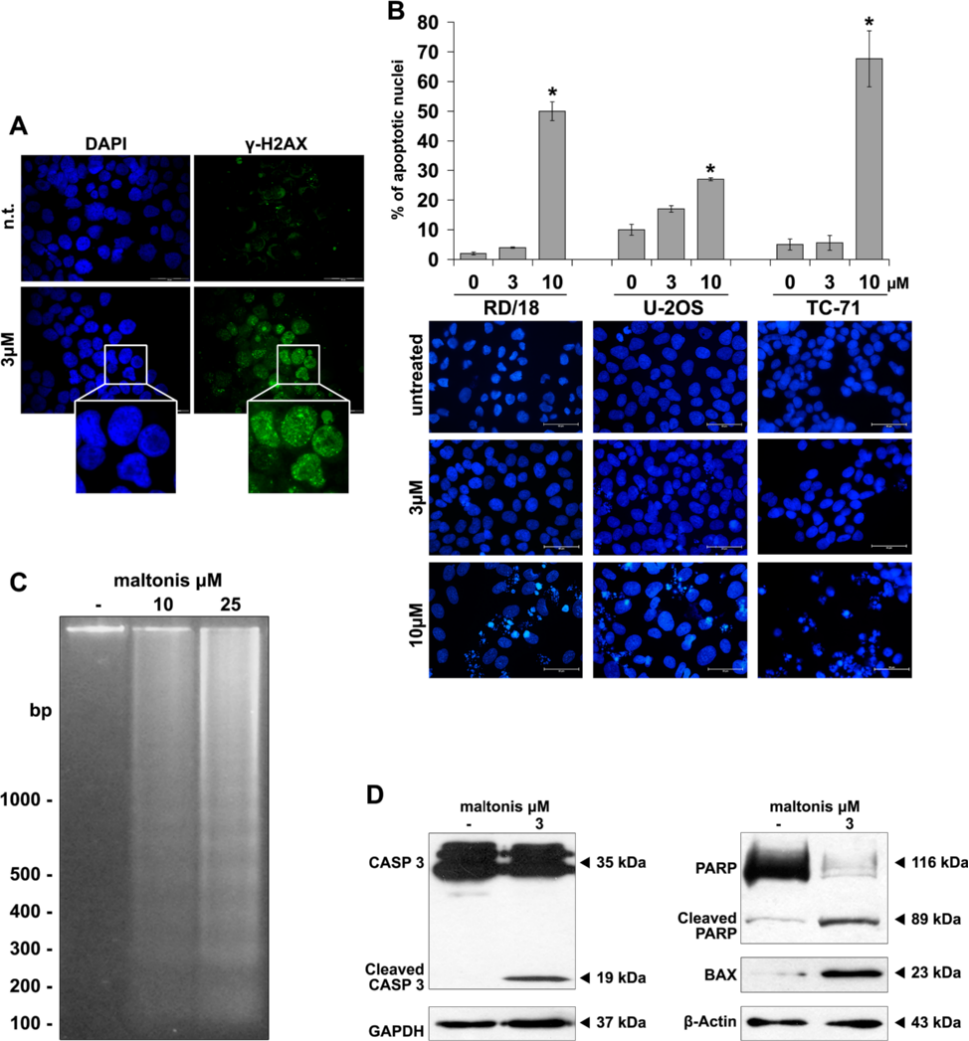
**Maltonis induces DNA damage and triggers apoptosis in sarcoma cell lines. (A)** Induction of H2AX phosphorylation (γ-H2AX) in TC-71 cells after exposure for 24 hours to 3 μM maltonis (magnification × 40) **(B)** Evaluation of nuclear fragmentation after staining with HOECHST-33258 on untreated or 3, 10 μM treated RD/18, U-2OS or TC-71 cells after 72 h. Histogram shows mean percentages ± SE of fragmented nuclei out of 1000 considered in three independent experiments. Statistical analysis was performed by Student’s t test: *P < 0.05. Representative pictures are also shown (magnification × 400). **(C)** Apoptosis-related DNA fragmentation induced by maltonis exposure after 72 hours at 10 or 25 μM in TC-71 cells. **(D)** Western blotting on TC-71 total cell lysates from control or maltonis treated cells (3 μM for 72 h) showing caspase 3 and PARP cleavage as well as BAX induction. Equal loading was monitored with anti-GAPDH or beta-actin blotting (lower lines).

We also assessed the phosphorylation of histone H2AX (γ-H2AX) as marker of DNA-damage response. TC-71 cells showed a precocious (treatments of 24 hours) increase of γ-H2AX levels when treated with maltonis at the concentration of 3 μM (Figure 4A, Additional file 6). In addition, the induction of apoptosis was confirmed morphologically by monitoring nuclear fragmentation after Hoechst staining (Figure 4B) and molecularly, at higher doses, by evaluating inter-nucleosomal cleavage and subsequent DNA laddering (Figure 4C). In TC-71 cells we were able to demonstrate PARP and Caspase 3 cleavage by western blotting after exposure to 3 μM maltonis (Figure 4D). Moreover, maltonis treatment was able to induce accumulation of BAX protein (Figure 4D).

### In vivo evaluation of maltonis efficacy against Ewing sarcoma xenografts

**Figure 5:**
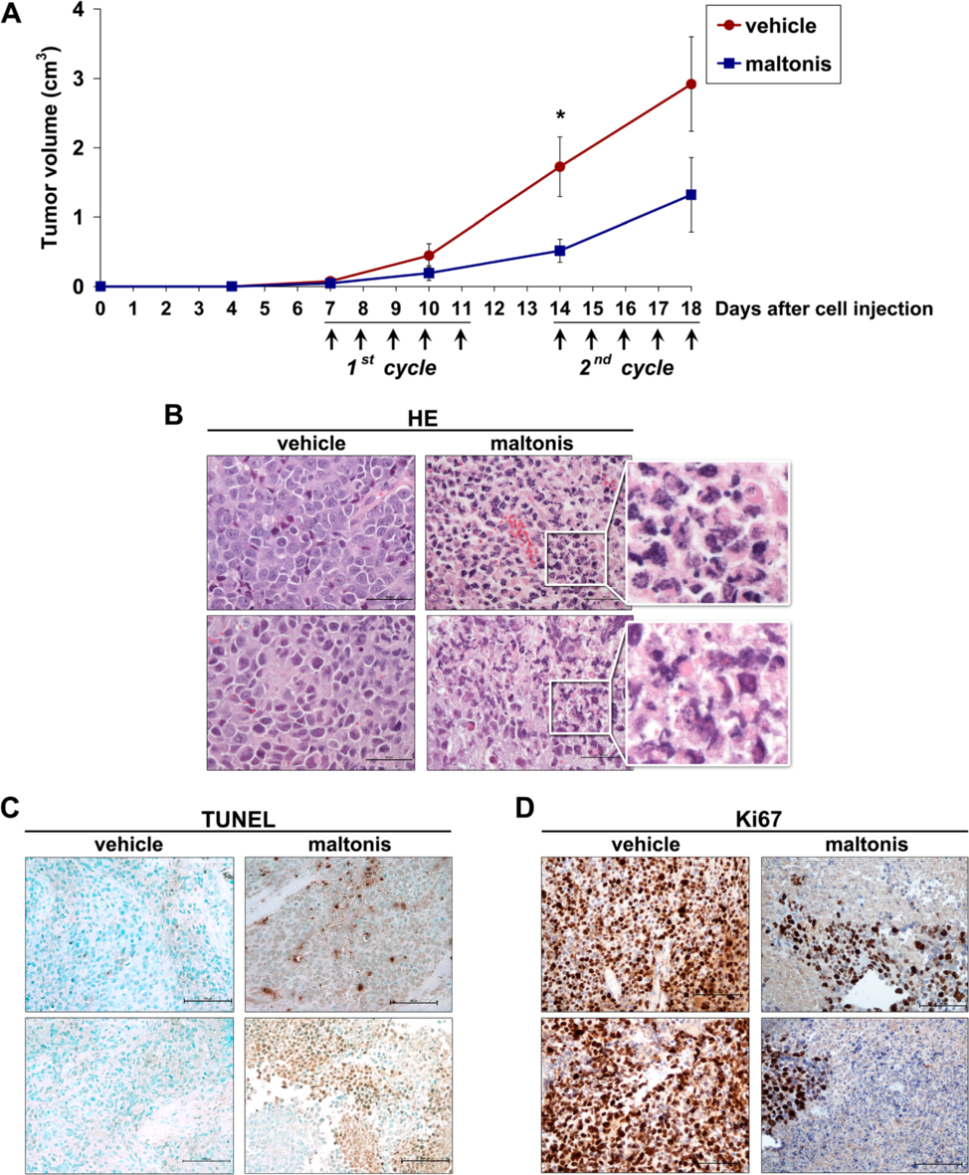
**Maltonis inhibits tumour growth and induces apoptotic cell death in xenograft model of sarcoma. (A)** Inhibition of TC-71 tumour growth in nude mice. Mice were treated for two cycles of 5 days with maltonis (1^st^ cycle: 20 mg/kg or 2^nd^ cycle: 40 mg/kg) or with vehicle (PBS). Panel shows mean tumour size ± SE of control or treated mice. **(B)** Morphologic hematoxylin-eosin of fragmented nuclei on tumour-samples derived from two controls (vehicle) or maltonis treated mice (magnification, ×400). **(C)** TUNEL assay evaluation of apoptotic nuclei after treatment with maltonis (magnification × 200. **(D)** Representative immunohistochemical evaluation of Ki67 in controls and maltonis treated tumours (magnification ×200).

**Figure 6:**
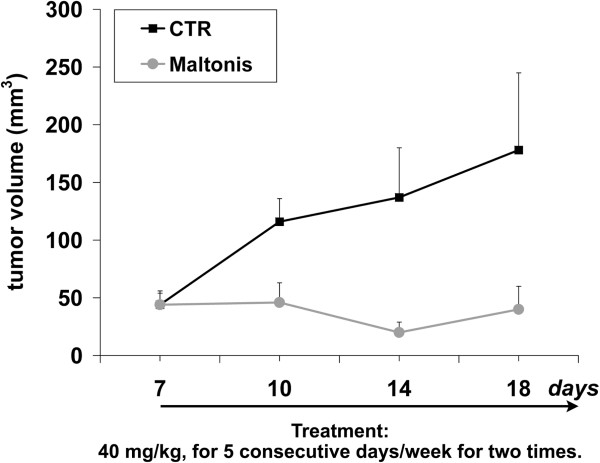
**Maltonis induces tumour inhibition in TC-71 xenografts.** Mice were treated for two cycles of 5 days with 40 mg/Kg of maltonis/week. Panel shows mean tumour size + SE of control or treated mice.

The anti-tumour activity of maltonis was evaluated in TC-71 xenograft model. TC-71 Ewing sarcoma cell line displays a very rapid cell growth *in vivo* and can therefore be representative of very aggressive sarcomas. In the first pilot experiment, we evaluated the therapeutic potential and toxicity of maltonis. Seven days after cell injection, when tumours began to be measurable, mice were divided into two subgroups: control mice were treated with vehicle alone (phosphate buffered saline), whereas treated mice received injection of maltonis, at increasing drug concentrations (20 mg/Kg in the first cycle and 40 mg/kg in the second one); four days after the end of the second cycle mice were sacrificed. Intra-tumour drug administration was chosen as first attempt in order to achieve higher local concentration and test only direct activity of the drug but not of its metabolites. At the indicated doses maltonis was very well tolerated: we did not observe any significant changes in mice weight, blood glucose and urea levels or hepatic enzymes activity (Additional file 7) or other sign of collateral toxicity. As reported in Figure 5A decrease in the growth rate was observed in maltonis-treated group (day 14, p value = 0.03; day 18, p value = 0.11, Mann Whitney test). Haematoxylin–eosin staining (HE) and TUNEL assay showed presence of apoptotic nuclei, featuring nuclear condensation and apoptotic body formation in treated samples (Figure 5B and C), sustaining the pro-apoptotic effects of maltonis. Evaluation of Ki67 from histological tissues of control and treated samples demonstrated that maltonis was also very effective in blocking tumour proliferation (Figure 5D) In the second experiment, mice were treated with the highest dose of maltonis (40 mg/kg for five consecutive days/week for two times). Growth inhibition was confirmed (Figure 6).

## Discussion

In this work we demonstrated that maltonis, a maltol derived compound, significantly reduces sarcoma cell viability and tumour growth either in monolayer and in anchorage-independent conditions while being basically ineffective on normal human mesenchymal stem cells. We also showed that maltonis was more effective in targeting sarcoma growth than its companion compound malten. Although previous chemical analysis indicated that malten should be more prone than maltonis to give non covalent approaches with negatively charged DNA [12], *in vitro* evaluation of impaired DNA properties (migration and amplificability) showed that maltonis induces a 9-fold higher amplification delay than malten, thus underlying a stronger perturbation of DNA structure which might be responsible for the major efficacy in sarcoma inhibition. Maltonis was found to inhibit cell proliferation and induce cell death. Accumulation of cells in G1 phase of cell cycle was observed in the U-2OS osteosarcoma and TC-71 Ewing cell lines, whereas in the rhabdomyosarcoma RD/18 model, accumulation was mainly in G2-M phase. In TC-71 the accumulation in G1 phase was coherent with the observed induction of p15 mRNA and increased p21 protein levels. Besides the cytostatic effect, maltonis was also able to deliver a cell death signal in all the three histotypes as demonstrated by flow cytometry analysis. Apoptosis was confirmed by nuclear fragmentation and detection of cleaved caspase 3 and PARP in TC-71 cells after exposure to the drug. The *in vitro* efficacy of this new compound was also confirmed *in vivo* against TC-71 Ewing sarcoma xenografts. Drug treatment produced a decrease in the growth rate of xenografts after treatment with maltonis in two separated independent experiments. Tumour volume reduction was likely due to both inhibition of cell proliferation and induction of apoptosis, thus substantially confirming what observed *in vitro*. Considering that maltonis activity has never been evaluated *in vivo* before, we could also provide evidence that the compound is well tolerated in mice at the highest and effective dose of 40 mg/kg.

Maltonis induces DNA fragmentation and recruits γH2AX [37,38] and Gadd45α [39], thus suggesting involvement of a DNA damage response. Similarly, the well characterized chemotherapeutic drug cisplatin has been recently reported to increase Gadd45α levels and to induce γH2AX foci [40,41]. Although the effects of maltonis and cisplatin could be interpreted as similar, the analysis of cisplatin-resistant variants indicated quite different mechanisms of action. Cells highly resistant to cisplatin (63 and 112 fold increase in resistance compared to sensitive parental cells) maintained a low level of residual resistance to maltonis (around 4 fold), thus suggesting that the two drugs most probably do not share common mechanisms of action and, therefore, do not undergo the same mechanisms of resistance [26]. Differently from cisplatin, that once inside the cell has to be aquated before being able to interact with DNA (and whose activity may thus be limited by interactions with endogenous detoxification molecules), maltonis is supposed to be able to enter the cell and to react directly with DNA. Physical interaction with the drug, demonstrated by cell free assays, is sufficient to alter nucleic acid properties and recruit the DNA damage repair system. This triggers characteristic biological effects, such as perturbation of cell cycle that culminate in activation of an irreversible cell death program. Considering that maltonis is not extruded by ABCB1, one of the major determinants of chemotherapeutic failure in osteosarcoma [42], this drug appears to be particularly interesting for a possible future treatment of sarcoma, offering an effective option for tumour inhibition also in refractory or resistant patients. A lesson learned from other neoplasms is that biologically disparate entities need subtype-specific treatments and therefore therapy should be designed in a disease-specific fashion according to the underlying biology. However rarity of sarcoma frequently couples with rarity of the target, making development of new, targeted drugs even less probable. Thus the identification of a new drug with effects similar to conventional chemotherapeutic agents, but exploiting different mechanisms of action and therefore active whenever doxorubicin, vincristine and cisplatin are ineffective, may overcome the economic and social problems of developing drugs for orphan diseases.

Overall, although further investigations are required in terms of dosage and schedule optimization, our findings propose maltonis as a potential candidate for the management of sarcomas. The designed synthetic procedure to obtain maltonis is quite simple and it does not contain hard multistep reactions. The products are obtained in good yield and already in a gram scale from commercial or well known starting molecules (maltol and 1,7-dimethyl-1,4,7,10-tetraazacyclododecane or 1,4-dimethyletylendiamine). This let us foresee an easy and low-cost synthetic scale up of the compound. Considering the almost complete absence of suitable option out of classical chemotherapeutic agents in the last two decades, maltonis might fulfill the need of new stable and easily synthesizable compounds effective especially for those refractory and metastatic sarcomas with poor outcome.

## Conclusions

This article demonstrates the efficacy of maltonis, a maltol-derived compound, on sarcomas and particularly on multidrug- and cisplatin-resistant cells, thus leading to the possible development of a new drug to be exploited in sarcoma patients both relapsed after first-line treatments or with metastasis at the diagnosis.

## Abbreviations

ROS: Reactive oxygen species; CDDP: Cisplatin; GSTP1: Glutathione S-transferase pi 1; NBDHEX: 6-(7-nitro-2,1,3-benzoxadiazol-4-ylthio)hexane; GAPDH: Glyceraldehyde 3-phosphate dehydrogenase; H2AX: Histone H2AX; ABCB1/MDR1: ATP-binding cassette sub-family B member 1/multidrug resistance protein 1; CDKI: Cyclin-dependent kinase inhibitors; CDKN2B: Cyclin-dependent kinase 4 inhibitor B; CDKN1A: Cyclin-dependent kinase inhibitor 1A; CDK6 and CDK8: Cyclin-dependent kinase 6 and 8; PARP: Poly (ADP-ribose) polymerase; BAX: BCL2-Associated X Protein; AST: Aspartate aminotransferase; ALT: Alanine aminotransferase.

## Competing interests

M. Fanelli e V. Fusi are listed as inventors in a patent application (WO2010/061282) submitted by the University of Urbino. All the other authors declare no competing interests.

## Authors’ contributions

KS and MF: experimental design, discussion of the data, manuscript writing and editing. CG: execution of the experimental plan, statistical elaboration of the data, ms writing and editing.MCM, AT, LG and MB: execution of the experimental plan, statistical elaboration of the data.SA: execution of the experimental plan, statistical elaboration of the data, manuscript editing.VF: design and molecules synthesis, discussion of the data, manuscript editing.LL, PLL: in vivo studies and elaboration of preclinical data. PP, MS and MMagnani: discussion of the data, manuscript editing; MManfrini: provision of material and discussion of data. LP: execution of experimental plan. All authors read and approved the final manuscript.

## Authors’ information

Katia Scotlandi and Mirco Fanelli shared senior-authorship.

## Pre-publication history

The pre-publication history for this paper can be accessed here:

http://www.biomedcentral.com/1471-2407/14/137/prepub

## Supplementary Material

Additional file 1 **List and sequences of primers employed for QPCR assay.***Description of data*: forward and reverse primers sets for all gene analysed in the QPCR assay to evaluate transcriptional modulation in maltonis treated cells. Click here for file

Additional file 2 **Synthetic pathway to obtain malten and maltonis.***Description of data*: Maltol (1), appropriately protected (2) and activated (3), was reacted with the polyamine (4) or (5) in THF in the presence of triethylamine (TEA) as a base. The treatment with 10% perchloric acid ethanol solution allows the simultaneous deprotection of the hydroxyl function and the cleansing of compounds thus avoiding tedious and expensive chromatographic purifications. Both compounds are obtained as hydroperchlorate white solid salt. Click here for file

Additional file 3 **Amplification delay (folds) for each set of primers as depicted in the “Primer Pair” column.***Description of data*: Amplification delay (folds) was calculated for each set of primers as the difference between the Ct values of treated and untreated samples. Click here for file

Additional file 4 **Effect of maltonis in anchorage independent-condition in a panel of sarcoma cell lines.***Description of data*: Representative images of anchorage independent growth of RMS, OS and ES with or without malten (30 μM) and maltonis (10 μM). Magnification × 40. Click here for file

Additional file 5 **Combined treatment with NBDHEX and CDDP or maltonis in cisplatin resistant osteosarcoma cell lines.***Description of data:* A dose of NBDHEX inhibiting GSTP1 activity but giving no growth inhibition was combined with increasing doses of CDDP or maltonis. Click here for file

Additional file 6 **Induction of H2AX phosphorylation (γ-H2AX) in TC-71 cells after 3 μM maltonis treatment.***Description of data:* confocal microscopy images of control or 3 μM maltonis treated TC-71 cells after 24 h exposure. Magnification × 63. Click here for file

Additional file 7 **Body mass and serum glucose, urea and transaminase levels in control and treated mice.***Description of data:* Before the sacrifice, mice were sampled for blood and determination of serum concentration of glucose and other enzymes was done. Moreover mean plus SE was calculated for mice weight in the two groups. Click here for file
