# Supplementary material for: An aza-macrocycle containing maltolic side-arms (maltonis) as potential drug against human pediatric sarcomas
Source: BMC Cancer. 2014 Feb 27;14:137. doi: 10.1186/1471-2407-14-137 (PMC3942616; doi:10.1186/1471-2407-14-137)
Supplement: Additional file 1 — List and sequences of primers employed for QPCR assay.Description of data: forward and reverse primers sets for all gene analysed in the QPCR assay to evaluate transcriptional modulation in maltonis treated cells. [file 1471-2407-14-137-S1.pdf]

GENE	PRIMER FORWARD	PRIMER REVERSE
**ABL1**	5'-TACCCGGGAATTGACCTGTC	5'-GGATTTCAGCAAAGGAGGGC
**ATM**	5'-GGTATAGAAAAGCACCAGTCCAGTATTG	5'-CGTGAACACCGGACAAGAGTTT
**ATR**	5'-AGTAGCTTCCTTTCGCTCCAAA	5'-ACTGACTCCGGCCACTCCAT
**BAX**	5'-CAAACTGGTGCTCAAGGCCC	5'-GGGCGTCCCAAAGTAGGAGA
**BCL2**	5'-CTGGTGGACAACATCGCCCT	5'-TCTTCAGAGACAGCCAGGAGAAAT
**BIRC5**	5'-CCGGTTGCGCTTTCCTTTC	5'-CGCACTTTCTCCGCAGTTTC
**BRCA1**	5'-GCATGCTGAAACTTCTCAACCA	5'-GTGTCAAGCTGAAAAGCACAAATGA
**BRCA2**	5'-AGACTGTACTTCAGGGCCGTACA	5'-GGCTGAGACAGGTGTGGAAACA
**CCNB1**	5'-ATGACATGGTGCACTTTCCTCC	5'-GCCAGGTGCTGCATAACTGG
**CCNB2**	5'-GATAACGAAGATTGGGAGAACCC	5'-CCACTAGGATGGCACGCATG
**CCNE1**	5'-AAATGGCCAAAATCGACAGG	5'-CGAGGCTTGCACGTTGAGTT
**CDC2**	5'-ACAGGTCAAGTGGTAGCCATGA	5'-ACCTGGAATCCTGCATAAGCA
**CDC16**	5'-ATGCTGAGGCCTTGGATTACC	5'-TCTCGCCTAAGACCAAGGGC
**CDC20**	5'-AGATGGACGACATTTGGCCA	5'-ATTGGACTGCCAGGGACACC
**CDC34**	5'-GGATTCCGCGTGACACTGGT	5'-ACCGAAAGGCTGGTGGAGAG
**CDK2**	5'-TTCTCATCGGGTCCTCCACC	5'-TCGGTACCACAGGGTCACCA
**CDK4**	5'-CTGTGCCACATCCCGAACTG	5'-GCCTCTTAGAAACTGGCGCA
**CDK6**	5'-CCGAAGTCTTGCTCCAGTCC	5'-GGGAGTCCAATCACGTCCAA
**CDK7**	5'-TCACATCTTCAGTGCAGCAGG	5'-TGGCAGCTGACATCCAGGT
**CDK8**	5'-AGCGGGTCGAGGACCTGTTT	5'-CATGCCGACATAGAGATCCCAG
**CDKN1A (p21)**	5'-TACCCTTGTGCCTCGCTCAG	5'-GGCGGATTAGGGCTTCCTCT
**CDKN1B (p27)**	5'-AGACTGATCCGTCGGACAGC	5'-CACAGAACCGGCATTTGGG
**CDKN2A (p14-p16)**	5'-CAACGCACCGAATAGTTACGG	5'-CTGCCCATCATCATGACCTG
**CDKN2B (p15)**	5'-ATCCCAACGGAGTCAACCG	5'-CTGCCCATCATCATGACCTG
**CDKN3**	5'-TGAAGCCGCCCAGTTCAATA	5'-CAACCTGGAAGAGCACATAAACC
**CHEK1**	5'-GAGCGTTTGTTGAACAAGATGTG	5'-GTTGGTCCCATGGCAATTCT
**CHEK2**	5'-TCAGCAAGAGAGGCAGACCC	5'-ACAGCTCTCCCCCTTCCATC
**CUL3**	5'-GGTAAACCAACACAGCGGGT	5'-CTGGGTCGGATTCACCTTGT
**E2F4**	5'-GCATCCAGTGGAAGGGTGTG	5'-ACGTTCCGGATGCTCTGCT
**GADD45A**	5'-GATGCCCTGGAGGAAGTGCT	5'-GAGCCACATCTCTGTCGTCGT
**KNTC1**	5'-ATAGTCAACCCAGAGTGGGCTGT	5'-TTTCACGTTTTTCGTCCTGCG
**MCM2**	5'-TGCCACTGTCATCCTAGCCA	5'-GATGGAAGGAGCAATGCTGG
**MKI67**	5'- TGTGCCTGCTCGACCCTACA	5'-TGAAATAGCGATGTGACATGTGCT
**PCNA**	5'-TTTGGTGCAGCTCACCCTG	5'-CGCGTTATCTTCGGCCCTTA
**RB1**	5'-GACCCAGAAGCCATTGAAATCT	5'-GGTGTGCTGGAAAAGGGTCC
**RPA3**	5'-TTCGTAGGGAGGCTGGAAAA	5'-CCTTGGCGGTTACTCTTCCAA
**TP53**	5'-GCGTGTTTGTGCCTGTCCTG	5'-TGGTTTCTTCTTTGGCTGGG
**UBE1**	5'-CCATAAACGCCTTCATTGGG	5'-TGGAGGCACTTGTCCTCTGTG
**GAPDH**	5'-GCAAATTCCATGGCACCGT	5'-TCGCCCCACTTGATTTTGG
