# Supplementary material for: An aza-macrocycle containing maltolic side-arms (maltonis) as potential drug against human pediatric sarcomas
Source: BMC Cancer. 2014 Feb 27;14:137. doi: 10.1186/1471-2407-14-137 (PMC3942616; doi:10.1186/1471-2407-14-137)
Supplement: Additional file 2 — Synthetic pathway to obtain malten and maltonis.Description of data: Maltol (1), appropriately protected (2) and activated (3), was reacted with the polyamine (4) or (5) in THF in the presence of triethylamine (TEA) as a base. The treatment with 10% perchloric acid ethanol solution allows the simultaneous deprotection of the hydroxyl function and the cleansing of compounds thus avoiding tedious and expensive chromatographic purifications. Both compounds are obtained as hydroperchlorate white solid salt. [file 1471-2407-14-137-S2.pdf]

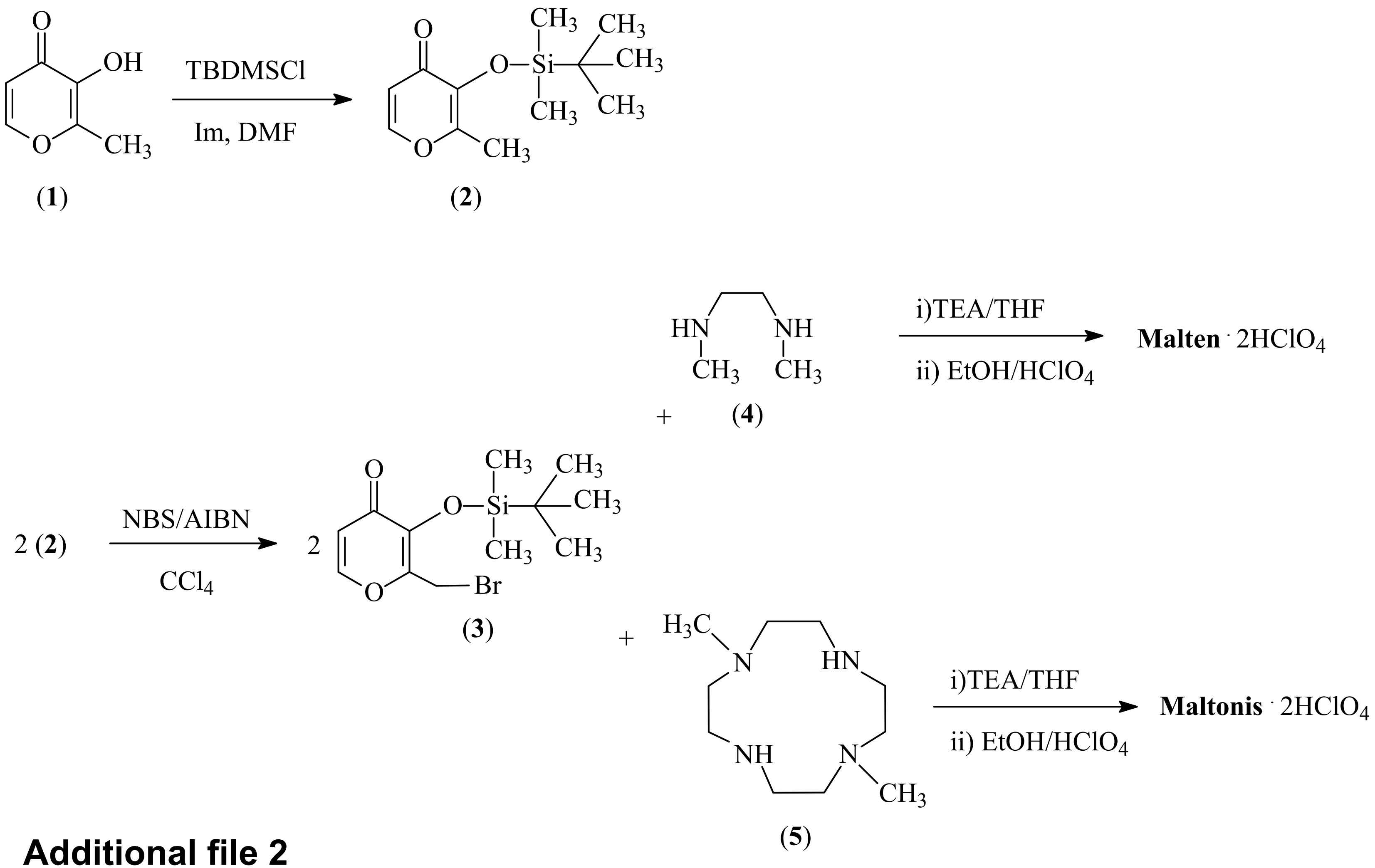

Additional file 2

Synthetic pathway to obtain Malten and Maltonis. Maltol (1), appropriately protected (2) and activated (3), was reacted with the polyamine (4) or (5) in THF in the presence of triethylamine (TEA) as a base. The treatment with 10% perchloric acid ethanol solution allows the simultaneous deprotection of the hydroxyl function and the cleansing of compounds thus avoiding tedious and expensive chromatographic purifications. Both compounds are obtained as hydroperchlorate white solid salt.
