# Supplementary material for: An aza-macrocycle containing maltolic side-arms (maltonis) as potential drug against human pediatric sarcomas
Source: BMC Cancer. 2014 Feb 27;14:137. doi: 10.1186/1471-2407-14-137 (PMC3942616; doi:10.1186/1471-2407-14-137)
Supplement: Additional file 3 — Amplification delay (folds) for each set of primers as depicted in the “Primer Pair” column.Description of data: Amplification delay (folds) was calculated for each set of primers as the difference between the Ct values of treated and untreated samples. [file 1471-2407-14-137-S3.pdf]

**Primer pairs** (amplicon lenght)	**Amplification delay** (folds)		
	**malten**	**maltonis**	**CDDP**
**F1→ 121 bp ←R**	**2.55**	**12.4**	**113**
**F2→ 179 bp ←R**	**4.5**	**59.5**	**643**
**F3→ 240 bp ←R**	**8.4**	**113**	**637**
**F4→ 301 bp ←R**	**23.7**	**212**	**1228**
**F5→ 479 bp ←R**	**206**	**1945**	**3449**
**F6→ 622 bp ←R**	**728**	**6119**	**4360**

Additional file 3
Amplification delay (folds) for each set of primers as depicted in the "Primer Pair" column.
