# Supplementary material for: An aza-macrocycle containing maltolic side-arms (maltonis) as potential drug against human pediatric sarcomas
Source: BMC Cancer. 2014 Feb 27;14:137. doi: 10.1186/1471-2407-14-137 (PMC3942616; doi:10.1186/1471-2407-14-137)
Supplement: Additional file 4 — Effect of maltonis in anchorage independent-condition in a panel of sarcoma cell lines.Description of data: Representative images of anchorage independent growth of RMS, OS and ES with or without malten (30 μM) and maltonis (10 μM). Magnification × 40. [file 1471-2407-14-137-S4.pdf]

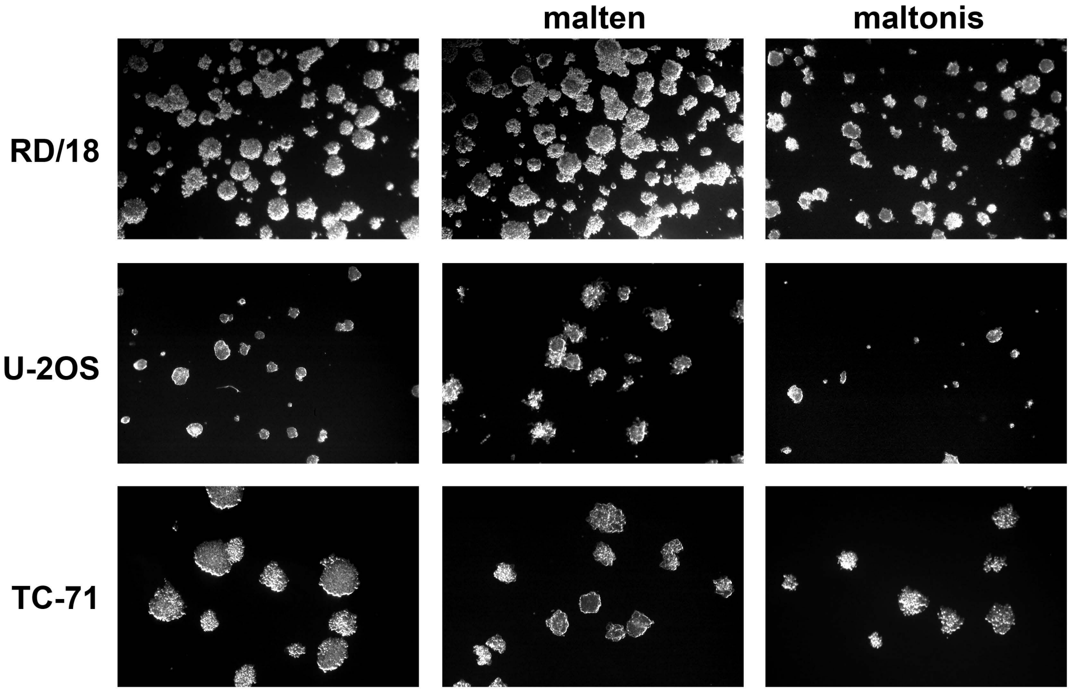

Additional file 4

Representative images of anchorage independent growth of RMS, OS and ES with or without malten (30μM) and maltonis (10μM). Magnification x40.
