# Supplementary material for: An aza-macrocycle containing maltolic side-arms (maltonis) as potential drug against human pediatric sarcomas
Source: BMC Cancer. 2014 Feb 27;14:137. doi: 10.1186/1471-2407-14-137 (PMC3942616; doi:10.1186/1471-2407-14-137)
Supplement: Additional file 5 — Combined treatment with NBDHEX and CDDP or maltonis in cisplatin resistant osteosarcoma cell lines.Description of data: A dose of NBDHEX inhibiting GSTP1 activity but giving no growth inhibition was combined with increasing doses of CDDP or maltonis. [file 1471-2407-14-137-S5.pdf]

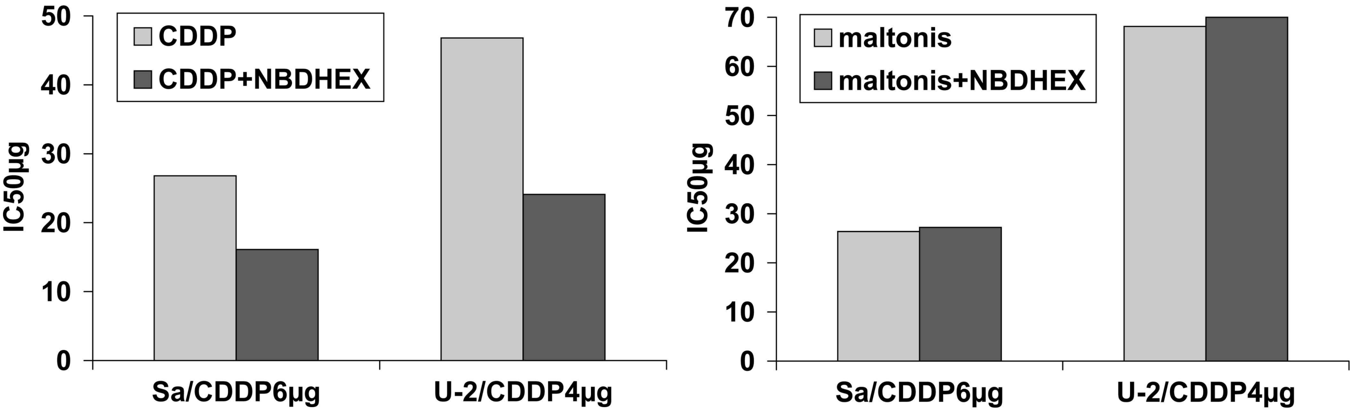

Additional file 5

combined treatment with NBDHEX and CDDP (panel A) or maltonis (panel B) in cisplatin resistant osteosarcoma cell lines. A dose of NBDHEX Inhibiting GSTP1 activity but giving no growth inhibition was combined with increasing doses of CDDP or maltonis. IC50, drug concentration resulting in 50% inhibition of cell growth.
