# Supplementary material for: An aza-macrocycle containing maltolic side-arms (maltonis) as potential drug against human pediatric sarcomas
Source: BMC Cancer. 2014 Feb 27;14:137. doi: 10.1186/1471-2407-14-137 (PMC3942616; doi:10.1186/1471-2407-14-137)
Supplement: Additional file 6 — Induction of H2AX phosphorylation (γ-H2AX) in TC-71 cells after 3 μM maltonis treatment.Description of data: confocal microscopy images of control or 3 μM maltonis treated TC-71 cells after 24 h exposure. Magnification × 63. [file 1471-2407-14-137-S6.pdf]

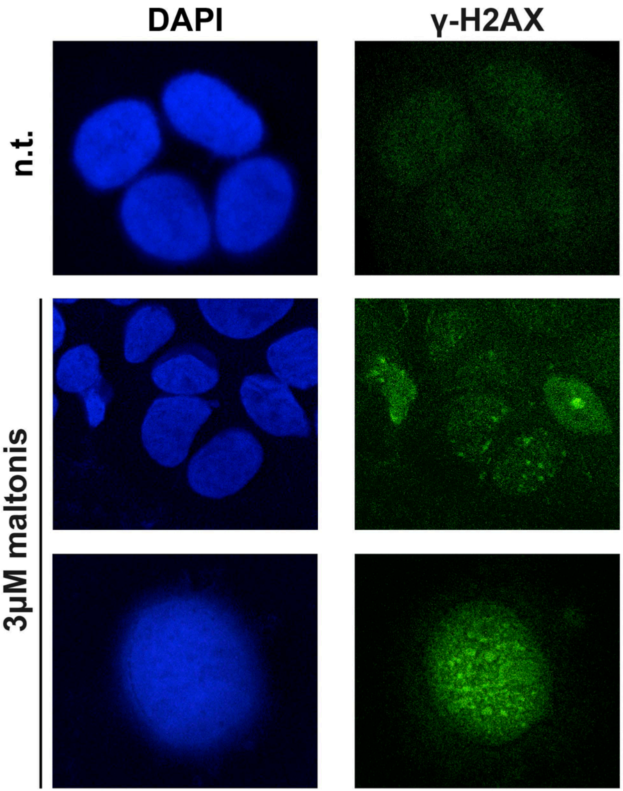

Additional file 6

Representative confocal images of γ-H2AX in control or maltonis treated (3μM) TC-71 with or without malten. Magnification x63.
