# Supplementary material for: An aza-macrocycle containing maltolic side-arms (maltonis) as potential drug against human pediatric sarcomas
Source: BMC Cancer. 2014 Feb 27;14:137. doi: 10.1186/1471-2407-14-137 (PMC3942616; doi:10.1186/1471-2407-14-137)
Supplement: Additional file 7 — Body mass and serum glucose, urea and transaminase levels in control and treated mice.Description of data: Before the sacrifice, mice were sampled for blood and determination of serum concentration of glucose and other enzymes was done. Moreover mean plus SE was calculated for mice weight in the two groups. [file 1471-2407-14-137-S7.pdf]

Samples	Mice weight (g)	Urea (mg/dL)	Glucose (mg/dL)	AST (IU/L)	ALT (IU/L)
**Control**	23.9 ± 0.43	51 ± 8	143 ± 32	121 ± 25	45 ± 10
**Maltonis**	23.3 ± 0.6	43 ± 6	123± 45	223 ± 111	58 ± 4

Additional file 7
Body mass and serum glucose, urea, and transaminase levels in control and treated mice. Abbreviations: AST, aspartate aminotransferase; ALT, alanine aminotransferase.

Note: Mice were sampled for blood at the end of the treatment (mean± se)
